# Comparative chromosome mapping of the rRNA genes and telomeric repeats in three Italian pine voles of the *Microtus savii* s.l. complex (Rodentia, Cricetidae)

**DOI:** 10.3897/CompCytogen.v5i3.1429

**Published:** 2011-08-24

**Authors:** Ekaterina Gornung, Alexandra M. R. Bezerra, Riccardo Castiglia

**Affiliations:** 1 Dipartimento di Biologia e Biotecnologie “Charles Darwin”, University of Rome “Sapienza”, via Alfonso Borelli 50, 00161, Rome, Italy; 2 Departamento de Zoologia, Universidade de Brasília, 70910–900, Brasília, DF, Brazil

**Keywords:** Arvicolinae, chromosomal evolution, sex chromosomes, interstitial telomeric sequences (ITS), rDNA, NORs

## Abstract

The *Microtus (Terricola) savii* *s. l.* complex is a group of five species/subspecies of the Italian pine voles, which diverged at different times either with or without chromosomal differentiation. The evidence of chromosomal diversification has so far concerned the shape of the sex chromosomes, especially the X chromosome. Three taxa of the group, *Microtus savii savii*, *Microtus savii nebrodensis*, and *Microtus savii tolfetanus* have identical karyotypes with metacentric X chromosomes. The X chromosomes of *Microtus brachycercus* and *Microtus brachycercus niethammericus* are, respectively, subtelocentric and acrocentric in shape. The *Microtus savii* complex has been long an object of conventional karyological studies, but comparative molecular cytogenetic data were completely missing. Therefore, we conducted a comparative chromosomal mapping of rRNA genes (rDNA) and telomeric repeats in three of the five taxa of the group: *Microtus savii savii*, *Microtus savii nebrodensis*, and *Microtus brachycercus niethammericus*, each of which belongs to a distinct mitochondrial clade.The survey revealed that differentiation of the clades was accompanied by remarkable changes with regard to the number and locations of the rDNA sites. Thus, *Microtus savii savii* and *Microtus savii nebrodensis* have especially high numbers of rDNA sites, which are located in the centromeric regions of, correspondingly, 18 and 13 chromosome pairs, whereas *Microtus brachycercus niethammericus* shows variable (8–10) and heteromorphic rDNA sites on both centromeric and telomeric regions. Interstitial telomeric sites (ITS), which are believed to indicate possible breakpoints of recurring chromosomal rearrangements, are present on the largest biarmed chromosomes and on the metacentric X chromosomes in *Microtus savii savii* and *Microtus savii nebrodensis*. These preliminary results are discussed in the context of recent advances in phylogeny of the group, as well as the rDNA genomic organization and X chromosome rearrangements in the genus *Microtus*.

## Introduction

The Italian endemic pine voles are distributed throughout the Apennine peninsula from the Alps to Sicily (Contoli et al. 2008). The chromosomal and morphological polytypism of this group led to the identification of five forms: “*savii”*, “*brachycercus”*, “*nebrodensis”*, “*niethammericus”*, and “*tolfetanus”*,ascribed to the *Microtus (Terricola) savii* complex de Sélys-Longchamps, 1838 (Galleni et al. 1994, 1998, Contoli 2003). The complex genetic structure found in the group (Castiglia et al. 2008) has not been found in the other species of the subgenus *Terricola* (Jaarola et al. 2004). The systematic ranks and relationships of these taxa have been recently reconsidered (Contoli 2008, Contoli and Nappi 2008) in the light of new insight into the phylogeny of the group (Castiglia et al. 2008) and chromosomal morphology (Table 1).

**Table 1.** The taxonomy and general karyological traits of Microtus savii s. l. complex

Old taxon1	New taxon2	2n, NFa	Sex chromosomes
***Microtus savii savii***	***Microtus savii savii***	**54, 58**	**X (m), Y (a)**
***Microtus savii nebrodensis***	Microtus savii nebrodensis*	**- “ -**	**- “ -**
*Microtus savii tolfetanus*	*Microtus savii tolfetanus*	- “ -	- “ -
*Microtus brachycercus*	*Microtus brachycercus*	- “ -	X (sm), Y (a)
***Microtus savii niethammericus***	***Microtus brachycercus niethammericus***	**- “ -**	**X (a), Y (a)**

m – metacentric, sm – submetacentric, a – small acrocentric, A – large acrocentric; * – assignment of species status is possible. 1 Musser and Carleton (2005), Contoli (2003); 2 Contoli (2008).

Karyological studies in the *Microtus savii* *sensu lato* complex revealed the same diploid number (2n=54) and invariable set of autosomes (NFa=58) in all these taxa, but only three of the five taxa showed sex chromosomes similar in size and shape. The sex chromosomes distinctiveness and the evidence of male sterility of hybrids between “*brachycercus*” and “*savii”* supporteda specific rank of “*brachycercus*”, which was first proposed by Galleni et al. (1994) and later accepted by Musser and Carleton (2005). The following analysis of phylogenetic relationships based on mitochondrial cytochrome *b* gene sequence variation revealed a strong similarity of haplotypes of “*brachycercus”* and *“niethammericus”* which indicated a possible co-specificity of the two taxa. Accordingly, Contoli and Nappi (2008) tentatively ascribed “*niethammericus”* to a subspecies of *Microtus brachycercus*. The difference in the shape of the X chromosomes of these two taxa was considered a polymorphism due to the paracentromeric heterochromatin accumulation (Castiglia et al. 2008), which is a common trend in many species of *Microtus* (see Marchal et al. 2004, Mitsainas et al. 2009). Finally, albeit the karyological similarity with *Microtus savii savii*, the Sicilian form presently ascribed to *Microtus savii nebrodensis* showed unexpectedly high genetic divergence, which suggested its possible specific status (Castiglia et al. 2008).

We further investigated the intra- and interspecific chromosomal variation in the Italian pine voles by analysing chromosomal distribution of rDNA and telomeric sequences. At present, we focused on three of the five taxa, i.e. most widespread and abundant *Microtus savii savii*, the Sicilian *Microtus savii nebrodensis*, and *Microtus brachycercus niethammericus*. Each of these taxa belongs to one of the three mitochondrial DNA clades identified in the group (Castiglia et al. 2008). The two taxa of the group, *Microtus savii tolfetanus* and *Microtus brachycercus brachycercus*, are missing from the present study. So far, comparative molecular cytogenetic data were not available in this interesting group of arvicoline rodents, which can possibly serve as a model to study chromosomal evolution.

## Materials and methods

Specimens of *Microtus savii savii* (two males and a female) were collected at three sites: Pizzone (Isernia, Molise), Parco dell’Appia and Passo Corese (Roma, Lazio). The specimens of *Microtus savii nebrodensis* (one male and one female) were trapped on the Nebrodi Mountains (Messina, Sicily). The individuals of *Microtus brachycercus niethammericus* (two males and one female) were trapped at Farindola (Pescara, Abruzzi). The animals were handled according to the European Code of Practice for the housing and care of animals used in scientific procedures (Council of Europe 1986). As a routine, metaphases were obtained from bone marrow using standard air-drying technique. 1 mg/ml Vinblastin sulfate (Velbe, Lilly) was used as a mitostatic agent. The karyotypes were analysed after standard Giemsa staining. C-banding (Sumner 1972) was performed mainly to discriminate between the acrocentric X chromosomes and the autosomes in *Microtus niethammericus*. In all specimens, two probes were used for FISH (Fluorescence In Situ Hybridization): 45S rDNA clone of *Xaenopus laevis*, biotin-labelled by random priming (Invitrogen, Life technologies), and a telomeric probe made of two complementary oligonucleotides (GGGTTA)7/(TAACCC)7 3’-end-labelled with biotin (M–Medical, Genenco). Standard procedures for hybridization of repetitive sequences were carried out (Lichter et al. 1992). Hybridization was followed by low-stringency (2xSSC/50% Formamide 1x3min; 2xSSC 3x5 min, RT) or high-stringency (1xSSC/50% Formamide at 40°C for 3 min; 2xSSC/50% Formamide at 40°C, 3x3 min; 2xSSC at 40°C, 3x3 min) post-hybridization washes; blocking with 3% BSA in 4xSSC, and three-round signal detection and amplification by Avidin-FITC/biotinylated anti-Avidin (Vector). Slides were customarily stained with propidium iodide and embedded in Vectashield medium (Vector). DAPI (4’, 6-diamidino-2-phenylindole) counterstaining facilitated identification of homologues after FISH. Digital images were acquired and elaborated by IPLab software (Photometrics) and then processed in Photoshop CS (Adobe Systems Inc., U.S.).

## Results

**Figure 1.**
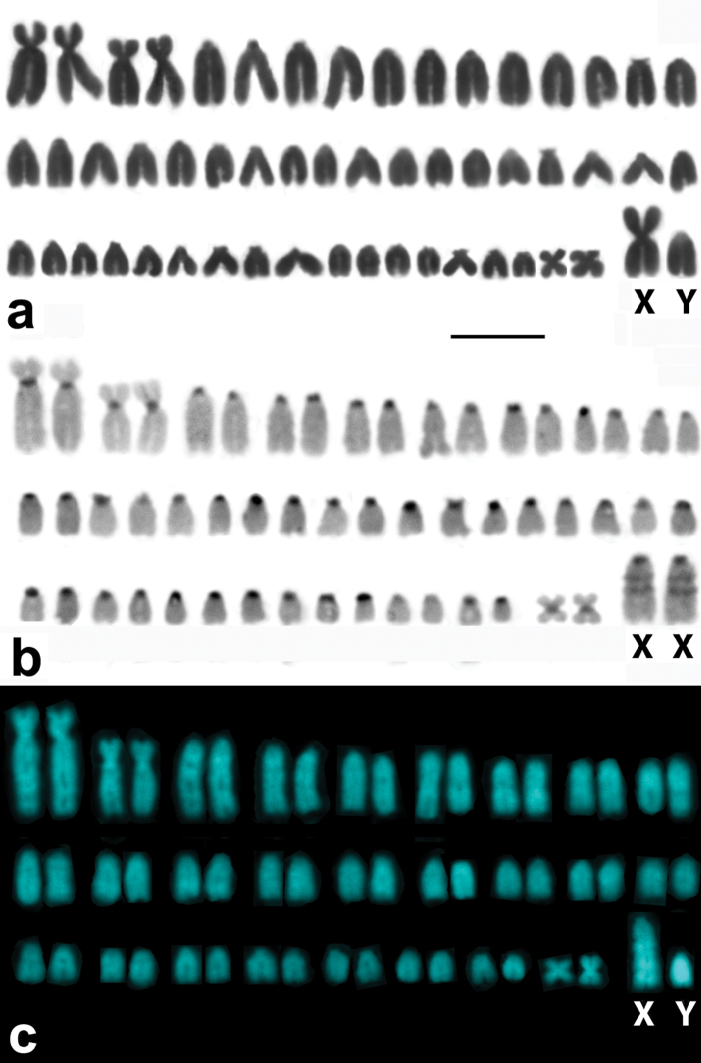
Representative karyotypes of the Italian pine voles. A conventional Giemsa stained male karyotype of a Savi’s pine vole exemplified by *Microtus savii nebrodensis* (**A**)with a large metacentric X chromosome and a small acrocentric Y. C-banded (**B**) and DAPI stained (**C**) chromosome complements of *Microtus brachycercus niethammericus*, which differ from (**A**) in morphology of the sex chromosomes. The large acrocentric X chromosomes of *Microtus brachycercus niethammericus* show distinctive prominent bands. *Bar* = 10 μm.

Specimens of *Microtus savii savii* and *Microtus savii nebrodensis* showed matching 2n=54 karyotypes with medium-size metacentric X and acrocentric Y chromosomes (Fig. 1a). Specimens of *Microtus brachycercus niethammericus* had a similar karyotype, but an acrocentric X chromosome of the same size as in the two other taxa. The characteristic C- and DAPI-banding patterns of this X chromosome (Fig. 1b, c) distinguished it from acrocentric autosomes.

**Figure 2.**
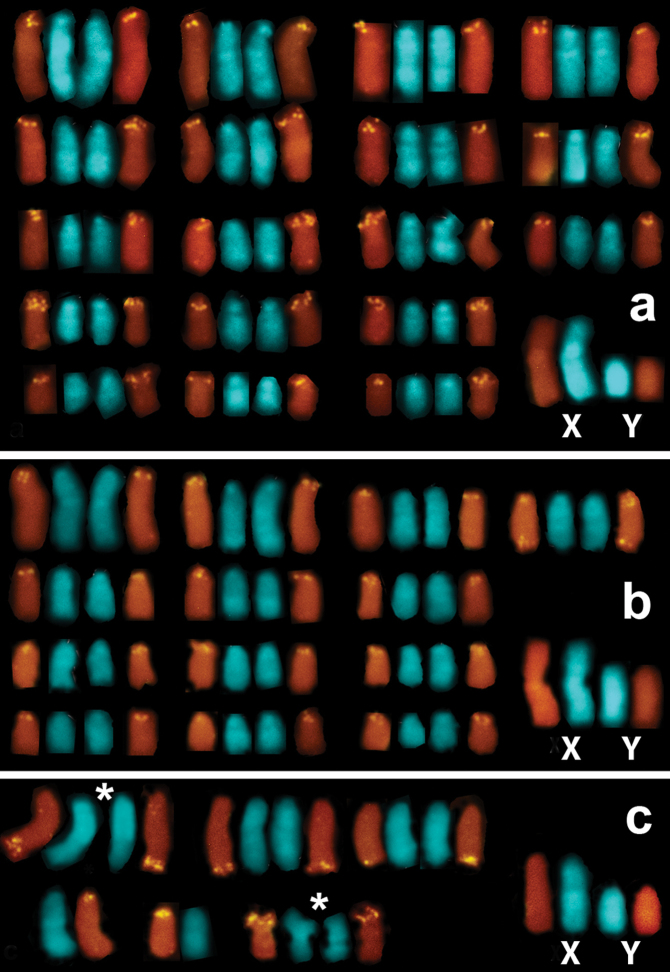
Partial karyotypes composed of rDNA-bearing chromosomes and the sex chromosomes of*Microtus savii savii* (**A**)*, M. s. nebrodensis* (**B**), and *Microtus brachycercus niethammericus* (**C**). The hybridization signals mark centromeric regions of all NOR-bearing chromosomes in *Microtus savii* subspecies (**A, B**) and, additionally, a telomeric region of a single chromosome in (**B**) (*upper row*). In (**C**), the largest among three individuals of *Microtus brachycercus niethammericus* set of rDNA-bearing chromosomes composed of two constantly marked chromosome pairs (signed by *asterisks*), one of which represent the smallest biarmed chromosomes, as well as chromosomes with variable rDNA sites, of which two chromosomes are in an apparently heterozygous state. The sex chromosomes lack rDNA-FISH signals in either subspecies.

Both the number and locations of the rDNA-FISH signals differed remarkably among the specimens (Fig. 2). The number of signal-bearing chromosomes in metaphase plates of *Microtus savii savii* was as large as 36 (18 chromosome pairs) (Fig. 2a), while 28 signals were distributed on 13 chromosome pairs in *Microtus savii nebrodensis* (Fig. 2b). The FISH signals were located at centromeres of acrocentric chromosomes in both Savi’s pine voles and only one pair of medium-sized acrocentric chromosomes of the Sicilian specimens was marked at both chromosome termini (Fig. 2b). The biarmed autosomes and the sex chromosomes lacked rDNA in both taxa (not illustrated). FISH revealed much lower number of rDNA sites per metaphase plate in *Microtus brachycercus niethammericus* (Fig. 2c). The overall rDNA-FISH pattern remained constant in different metaphase cells of each individual of *Microtus brachycercus niethammericus*, but differed slightly among presently studied individuals (8, 9 and 10 signals per cell). In this species, hybridization signals were constantly present on two distinct pairs of homologues, a pair of medium-sized acrocentric chromosomes and the smallest pair of metacentric chromosomes, whereas the remaining 4–6 FISH signals were detected on a small set of apparently non-homologous chromosomes. This pattern persisted under various hybridization conditions.

**Figure 3.**
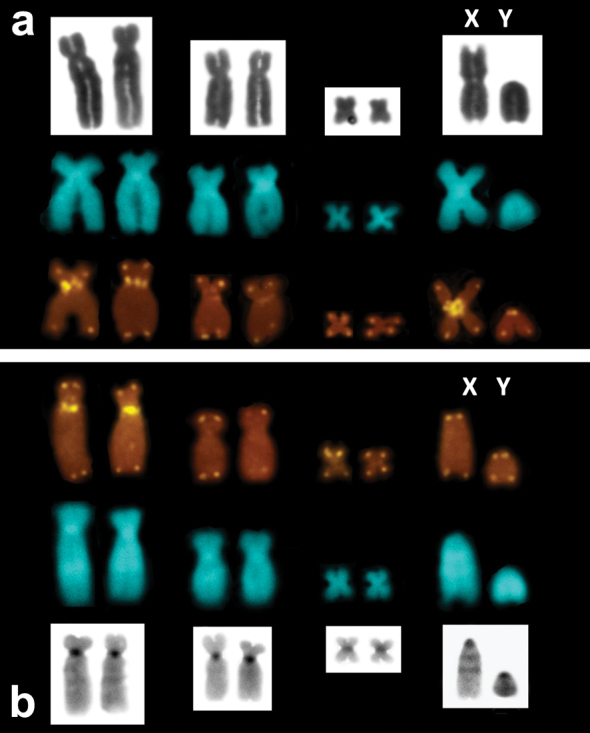
Telomeric FISH signals on thebiarmed and the sex chromosomes of *Microtus savii*, exemplifiedby *Microtus savii savii* (**A**) and*Microtus brachycercus niethammericus* (**B**). Respective chromosome pairs are shown after DAPI counterstaining (*central row* in **A, B**), Giemsa staining (*upper row in* **A)** and after C-banding (*lower row* in **B**). ITS are present on the largest biarmed chromosomes and on the metacentric X chromosome.

FISH with the telomeric probe followed by high-stringency post-hybridization washes (PHW) showed an ordinary, all-telomeric, pattern in all the specimens studied. Nonetheless, by decreasing the stringency of PHW we revealed telomeric-like sequences on some chromosomes. Thus, prominent ITS signals were present in the centromeric regions of the metacentric X and the largest biarmed (submetacentric) chromosomes of *Microtus savii savii* and *Microtus savii nebrodensis* (Fig. 3a). Other chromosomes including two of the three pars of biarmed autosomes (submetacentric and tiny metacentric) and the Y chromosome did not show telomeric signals even under low-stringency conditions. In *Microtus brachycercus niethammericus*, FISH detected the same ITS pattern on the largest biarmed chromosomes, but the acrocentric X chromosome lacked any interstitial signal (Fig. 3b).

## Discussion

The study revealed a considerable variation in the number and chromosomal distribution of rDNA sites at both intraspecific (between *Microtus savii savii* and *Microtus savii nebrodensis*)and interspecific (between the two Savi’s pine voles and *Microtus brachycercus niethammericus*) levels.

The two Savi’s pine voles, *Microtus savii savii* and *Microtus savii nebrodensis*, separated in middle-early Pleistocene (0.6–1.0 MYA) (Castiglia et al. 2008) have almost identical karyotypes and share the same general rDNA pattern, i.e. an abundant number of rDNA sites, their chiefly centromeric locations, and absence on the sex chromosomes. The intraspecific differences between the two Savi’s pine voles concern the exact number of rDNA-bearing chromosome pairs, 13 in *Microtus savii nebrodensis* and 18 in *Microtus savii savii*, and the presence of additional telomeric signals on one chromosome pair of *Microtus savii nebrodensis*.

The rDNA pattern of *Microtus brachycercus niethammericus* differs peculiarly from the ones of the congeneric species. The mean number of rDNA sites is markedly lower. The sites appear variable in number and size and are located in both centromeric and telomeric regions of a small set of chromosomes. This evidence is in accord with the genetic divergence of “*brachycercus”* clade separated from *Microtus savii savii* in middle Pleistocene (0.3–0.5 MYA) (Castiglia et al. 2008).

The number and chromosomal locations of NORs (nucleolar organizer regions, rDNA sites) have been comprehensively studied in various species of the genus *Microtus* by conventional silver staining technique (Zagorodnyuk 1992, Mazurok et al. 1996, Mazurok et al. 2001, Mekada et al. 2001, Martínková et al. 2004). Even if this method reveals not all rDNA sites, but the NORs, which were transcriptionally active in the previous interphase (Ag-NORs), the records reflect a remarkable interspecific variation of NORs in the genus. The putative ancestral karyotype of the genus *Microtus* is considered to be 2n=54 (Zima and Král 1984, Martínková et al. 2004, Lemskaya et al. 2010). Analysing the Ag-NORs data, we noted that the species of *Microtus* with derived karyotypes commonly show minor numbers of NORs (Gornung et al. 2011). In contrast, the species with primitive karyotypes, such as *Microtus rossiaemeridionalis* (2n=54, NFa=54) (Mazurok et al. 1996) or *Microtus transcaspicus* (2n=52, NFa=52) (Mazurok et al. 2001), have numerous NORs (up to 16 NOR-bearing chromosome pairs in *Microtus rossiaemeridionalis*) predominantly located in the centromeric regions of chromosomes.

The increase in the number of NORs in the evolution of different groups of species, so-called rDNA dispersion, is well documented. Reciprocal translocations at the level of C bands are supposed to be the basic underlying mechanism of this event (Hirai et al. 1996). Accordingly, the location of NORs in the C-positive centromeric regions facilitates the dispersion. Moreover, as recently proposed for the genus *Mus*, the accumulation of a large number of rDNA repeats in the centromeric region may represent an important first step of chromosome re-patterning, which may be triggered by modifications of the epigenetic state of DNA (Cazaux et al. 2011). Regardless of a possible mechanism of remodelling of NORs patterns, such as aforementioned translocation events, unequal crossing over or transposition with subsequent amplification of rDNA (Eickbush and Eickbush 2007), the present data imply the evolutionary genomic plasticity of the *Microtus savii* group.

Like in all species of *Microtus* thus studied, except *Microtus kirgisorum* (Mazurok et al. 2001), NORs have not been detected on the sex chromosomes in the three presently studied taxa. The sex chromosomes of several species of *Microtus* show complex and heterogeneous heterochromatin, which is indicative of a rapid turnover of repetitive sequences in the genus (Modi 1987, Burgos et al. 1988, Marchal et al. 2004). In the *Microtus savii* complex, only satellite DNA Msat-160 has been described (Acosta et al. 2010). According to these data, despite a similar autosomal distribution, the amount of Msat-160 in the pericentromeric regions ofchromosomes, including those of the X chromosome, in *Microtus brachycercus niethammericus* is clearly lower than in *Microtus savii savii*. Moreover, Msat-160 is present on the Y chromosome of *Microtus brachycercus niethammericus*, but absent on the Y of *Microtus savii*. In addition, the report of Galleni et al. (1992) described particular *Alu*I bands on the X chromosomes of *Microtus brachycercus*.

Presently, we show that while interstitial telomeric-like sequences are marking the largest pair of biarmed chromosomes in either species, they are also present in the centromeric region of the metacentric X chromosome of *Microtus savii savii* and *Microtus savii nebrodensis*, whereas absent in the heterochromatic regions of the acrocentric X chromosome of *Microtus brachycercus niethammericus*. We hypothesize that according to the basal position of *Microtus savii nebrodensis* in the phylogenetic reconstruction (Castiglia et al. 2008) the metacentric X chromosome should be primitive in the group. It follows that the other forms of the X chromosome found in *Microtus brachycercus niethammericus* and *Microtus brachycercus brachycercus* may have originated by pericentric inversion of the ancestral metacentric X with subsequent amplification of pericentromeric heterochromatin in *Microtus brachycercus brachycercus*. In view of the fact that clusters of different repetitive DNA including subtelomeric and interstitial telomeric repeats characterize the breakpoints of recurrent chromosomal rearrangements (Azzalin et al. 2001, Nergadze et al. 2004), the presence of interstitial telomeric-like DNA sequences in the presumably primitive metacentric X chromosome is dispuwle. Indeed, according to the evolutionary relationships in a group of voles of another subgenus, *Microtus*, the metacentric X chromosome was supposed to be “derived” respect to the acrocentric and submetacentric morphology (Mazurok et al. 2001,Nesterova et al. 1998). On the other hand, the X chromosomes might have undergone sequential inversions in the chromosomal evolution of the genus. Several different breakpoints were indeed identified on the X chromosomes of some *Microtus* species (Rubtsov et al. 2002).

To date, the evidence of chromosomal diversification in the *Microtus savii* s.l. complex concerned only the shape of the sex chromosomes, particularly the X chromosome. Presently, we can add several details to this evidence and conclude that significant changes of rDNA genomic organization accompanied the genetic differentiation of the Italian pine voles.
